# Commentary: Discrepancies Between Explicit Feelings of Power and Implicit Power Motives Are Related to Anxiety in Women With Anorexia Nervosa

**DOI:** 10.3389/fpsyg.2021.670436

**Published:** 2021-05-25

**Authors:** Oliver C. Schultheiss

**Affiliations:** Department of Psychology, Friedrich-Alexander University, Erlangen, Germany

**Keywords:** implicit motives, assessment, critique, self-attributed needs, declarative measures, motivation

## Introduction

In their paper, Weineck and colleagues claim to have measured implicit power motives with the Multi-Motive Grid (MMG), an instrument that features pictures of ambiguous social situations and asks respondents to judge each according to a series of declarative statements that may characterize the needs, feelings, or actions of the depicted persons as true or false. Notably, the MMG was not labeled a measure of implicit motives in the original publication (Sokolowski et al., 2000). But subsequent research portrayed the MMG as a measure of implicit motives (e.g., Kehr, 2004; Schüler et al., 2015), and two of the MMG's original authors eventually did, too (Langens and Schmalt, 2008). However, grid-type measures like the MMG *do not* assess implicit motives (Schultheiss and Köllner, 2021). Rather, they appear to assess constructs closely related to self-attributed (or explicit) motives. I base this conclusion on the following observations:

1. Grid-type measures have not been shown to be sensitive to variations in motivational states

Adopting principles of valid measurement from the natural sciences, McClelland (1958, 1987) made it a central criterion for the validity of motive measures that they be sensitive to variations in motivational arousal (see also Borsboom et al., 2004). He and his colleagues showed this criterion to hold for motive measures based on stories respondents tell about ambiguous picture cues (Atkinson, 1958; Smith, 1992). Such sensitivity for variations in motivational arousal was never systematically documented for the MMG or other grid-type measures. Hence, we know nothing about the causal processes generating variations in scores on such measures. This is the most fundamental problem with grid-type measures purporting to measure motivational needs (see also Boag, 2015).

2. Grid-type measures do not converge with validated implicit motive measures

Sokolowski et al. (2000) failed to provide evidence for a convergence between the MMG and story-telling measures of implicit motives validated in the manner described above. Subsequent research aiming to address this lacuna with adequately powered samples has consistently failed to document systematic and substantial positive correlations between grid-type measures and implicit motive measures. For instance, Schüler et al. (2015; *N* = 202) found the MMG scales for achievement, affiliation, and power to correlate with corresponding measures on a standard picture-story motive measure only at −0.12, 0.14, and 0.09, respectively, representing less than 2% of shared variance. Similar results were obtained by Brunstein and Heckhausen (2008; *N* = 220) for the MMG and story-telling measures of achievement motivation and by Schultheiss et al. (2009; Schüler et al. (2015; *N* = 195; also cited by Weineck et al., 2021) for a grid-type measure matched even more closely than the MMG to the standard picture-story motive measure (see also Neumann and Schultheiss, 2015). More broadly, these findings are consistent with the meta-analytical observation of near-zero correlations between picture-story motive measures and measures trying to assess motives through declarative statements (see Schultheiss, 2007; Köllner and Schultheiss, 2014).

3. Grid-type measures converge with other measures of self-attributed (explicit) motives and traits

Across several published studies, findings support the view that the MMG and similar measures show a moderate degree of positive overlap with questionnaire-based motive measures, although not always in a manner in which the strongest convergence emerges for the diagonal elements of multimethod correlation matrices. For instance, Kehr (2004, *N* = 82) found the MMG achievement scale to correlate 0.28, 0.21, and 0.28, respectively, with the Personality Research Form (PRF; Jackson, 1984) scales achievement, affiliation, and dominance, and obtained similarly scattered correlations for the MMG scales affiliation and power. Likewise, Schultheiss et al. (2009); (*N* = 190) reported correlations of their grid-type power motive scale with the PRF scales dominance, aggression, achievement, and affiliation of 0.20, 0.23, 0.02, and 0.17, respectively. Langens et al. (2005); (*N* = 94) reported convergence coefficients of the MMG scales for achievement, affiliation, and power with the corresponding PRF scales of 0.22, 0.23, and 0.22, respectively. Such findings suggest that the MMG and related measures tap into individuals' explicit, self-attributed motives (see Schultheiss et al., 2009; Schultheiss and Köllner, 2021). Also note that measures combining pictures with declarative items have been used to assess traits; they converge strongly with classic trait measures based on self-report items only (Paunonen et al., 2001).

## Conclusion

It may be relevant to explore, as Weineck and colleagues did, to what extent women with anorexia nervosa endorse statements expressing fear of power or hope for power. It may also be relevant how these statements relate to such individuals' feelings of power. But researchers should not invoke concepts and theories of implicit motives in general and implicit power motivation in particular to frame and explain such observations if they use measures that have no empirically well-delineated relationship with these concepts and theories. Thus, the question of what the observed findings for women with anorexia nervosa signify cannot be satisfactorily answered, and implications for clinical treatments should not be inferred, based on the approach chosen by Weineck et al. (2021).

More generally, progress in motivational accounts of disorders such as anorexia nervosa is unlikely to occur if motive measures with unclear validity are used in research and those with better validity credentials, such as story-telling measures (for a possible alternative see Slabbinck et al., 2011) are omitted. The fundamental difference between these two types of measures has been obscured by declaring one to be equivalent to the other. As McClelland et al. (1989) stated more than 30 years ago about the missing convergence between declarative and story-telling motive measures: “Another way to react to this lack of correlation is to take it seriously, to insist that at a minimum, psychologists should not call by the same name two measures that do not correlate with one another […]” (p. 691).

## Author Contributions

The author confirms being the sole contributor of this work and has approved it for publication.

## Conflict of Interest

The author declares that the research was conducted in the absence of any commercial or financial relationships that could be construed as a potential conflict of interest.

## References

[B1] Atkinson J. W. (1958). Motives in Fantasy, Action, and Society: A Method of Assessment and Study. Princeton, NJ: Van Nostrand.

[B2] Boag S. (2015). Personality assessment, ‘construct validity', and the significance of theory. Pers. Individ. Diff. 84, 36–44. 10.1016/j.paid.2014.12.039

[B3] Borsboom D. Mellenbergh G. J. van Heerden J. (2004). The concept of validity. Psychol. Rev. 111, 1061–1071. 10.1037/0033-295X.111.4.1061 15482073

[B4] Brunstein J. C. Heckhausen H. (2008). Achievement motivation, in Motivation and Action, 2 Edn, eds H. Heckhausen and J. Heckhausen (New York, NY: Cambridge University Press), 137–183. 10.1017/CBO9780511499821.007

[B5] Jackson D. N. (1984). Personality Research Form, 3 Edn. Port Huron, MI: Sigma Assessment Systems, Inc.

[B6] Kehr H. M. (2004). Implicit/explicit motive discrepancies and volitional depletion among managers. Pers. Soc. Psychol. Bull. 30, 315–327. 10.1177/0146167203256967 15030623

[B7] Köllner M. Schultheiss O. C. (2014). Meta-analytic evidence of low convergence between implicit and explicit measures of the needs for achievement, affiliation, and power. Front. Psychol. 5:826. 10.3389/fpsyg.2014.00826 25152741 PMC4126572

[B8] Langens T. Schmalt H.-D. Sokolowski K. (2005). Motivmessung: Grundlagen und Anwendungen [Motive assessment: Basic principles and applications], in Motivationspsychologie und ihre Anwendung, 3 Edn, eds R. Vollmeyer and J. Brunstein (Thousand Oaks, CA: Kohlhammer), 72–91.

[B9] Langens T. A. Schmalt H.-D. (2008). Motivational traits: new directions and measuring motives with the multi-motive grid (MMG), in The SAGE Handbook of Personality Theory and Assessment, Vol 1: Personality Theories and Models (Stuttgart: Sage Publications, Inc.), 523–544. 10.4135/9781849200462.n25

[B10] McClelland D. C. (1958). Methods of measuring human motivation, in Motives in Fantasy, Action, and Society: A Method of Assessment and Study, ed J. W. Atkinson (Princeton, NJ: Van Nostrand), 7–42.

[B11] McClelland D. C. (1987). Human Motivation. New York, NY: Cambridge University Press.

[B12] McClelland D. C. Koestner R. Weinberger J. (1989). How do self-attributed and implicit motives differ? Psychol. Rev. 96, 690–702. 10.1037/0033-295X.96.4.690

[B13] Neumann M.-L. Schultheiss O. C. (2015). Implicit motives, explicit motives, and critical life events in clinical depression. Cogn. Ther. Res. 39, 89–99. 10.1007/s10608-014-9642-8

[B14] Paunonen S. V. Ashton M. C. Jackson D. N. (2001). Nonverbal assessment of the big five personality factors. Eur. J. Pers. 15, 3–18. 10.1002/per.385

[B15] Schüler J. Brandstätter V. Wegner M. Baumann N. (2015). Testing the convergent and discriminant validity of three implicit motive measures: PSE, OMT, and MMG. Motiv. Emot. 39, 839–857. 10.1007/s11031-015-9502-1

[B16] Schultheiss O. C. (2007). A memory-systems approach to the classification of personality tests: comment on Meyer and Kurtz (2006). J. Pers. Assess. 89, 197–201. 10.1080/00223890701357431 17764396

[B17] Schultheiss O. C. Köllner M. G. (2021). Implicit motives, in Handbook of Personality: Theory and Research, 4 Edn, eds O. P. John and R. W. Robins (New York, NY: Guilford), 385–410.

[B18] Schultheiss O. C. Yankova D. Dirlikov B. Schad D. J. (2009). Are implicit and explicit motive measures statistically independent? A fair and balanced test using the Picture Story Exercise and a cue- and response-matched questionnaire measure. J. Pers. Assess. 91, 72–81. 10.1080/00223890802484456 19085286

[B19] Slabbinck H. De Houwer J. Van Kenhove P. (2011). A pictorial attitude IAT as a measure of implicit motives. Eur. J. Pers. 25, 76–86. 10.1002/per.778

[B20] Smith C. P. (ed.). (1992). Motivation and Personality: Handbook of Thematic Content Analysis. New York, NY: Cambridge University Press.

[B21] Sokolowski K. Schmalt H. D. Langens T. A. Puca R. M. (2000). Assessing achievement, affiliation, and power motives all at once: the Multi-Motive Grid (MMG). J. Pers. Assess. 74, 126–145. 10.1207/S15327752JPA740109 10779937

[B22] Weineck F. Schultchen D. Dunker F. Hauke G. Lachenmeir K. Schnebel A.. (2021). Discrepancies between explicit feelings of power and implicit Power motives are related to anxiety in women with anorexia nervosa. Front. Psychol. 11:618650. 10.3389/fpsyg.2020.618650 33633629 PMC7901641

